# Effects of the Mental Health and Welfare Law revision on schizophrenia patients in Korea: an interrupted time series analysis

**DOI:** 10.1186/s13033-021-00499-3

**Published:** 2021-10-14

**Authors:** Jongho Heo, Nan-He Yoon, Soyoun Shin, Soo-Young Yu, Manwoo Lee

**Affiliations:** 1 National Assembly Futures Institute, National Assembly Member’s Hall, 1, Uisadang-daero, Yeongdeungpo-gu, Seoul, 07233 Republic of Korea; 2 Division of Social Welfare and Health Administration, Wonkwang University, 460 Iksandae-ro, Iksan, 54538 Republic of Korea; 3 Seoul National University College of Nursing Seoul, 103 Daehak-ro, Jongno-gu, Seoul, 03080 Republic of Korea; 4 Department of Nursing Science, Jeonju University, 303, Cheonjam-ro, Wansan-gu, Jeonju, Jeollabuk-do, 55069 Republic of Korea; 5 National Assembly Research Service, 1, Uisadang-daero, Yeongdeungpo-gu, Seoul, 07233 Republic of Korea

**Keywords:** Involuntary hospitalization, South Korea, Re-admission, Human rights

## Abstract

**Background:**

High rates of involuntary hospitalization and long lengths of stay have been problematic in Korea. To address these problems, the Mental Health and Welfare Law was revised in 2016, mainly to protect patient rights by managing involuntary admissions. The aim of this study was to evaluate the impact of the revised Mental Health and Welfare Law on deinstitutionalization by using routinely collected data from hospital admissions and continuity of mental health service use after hospital discharge as proxy measures of deinstitutionalization.

**Methods:**

We used monthly-aggregated claims-based data with a principal or secondary diagnosis of schizophrenia from 2012 to 2019, collected by the National Health Insurance Service. Outcome variables included rates of first admission; discharges; re-admissions within 7, 30, and 90 days; outpatient visits after discharge within 7 and 30 days; and continuity of visits, at least once a month for 6 months after discharge. Using interrupted time series analysis, we estimated the change in levels and trends of the rates after revision, controlling for baseline level and trend.

**Results:**

There was no significant change in first admission and discharge rates after the revision. Immediately after the revision, however, the rates of re-admission within 7 and 30 days dropped significantly, by 2.24% and 1.99%, respectively. The slopes of the re-admission rate decreased significantly, by 0.10% and 0.14%, respectively. The slopes of the re-admission rate within 90 days decreased (0.001%). The rates of outpatient visits within 7 and 30 days increased by 1.98% and 2.72%, respectively. The rate of continuous care showed an immediate 4.0% increase.

**Conclusions:**

The revision had slight but significant effects on deinstitutionalization, especially decreasing short-term re-admission and increasing immediate outpatient service utilization.

## Introduction

Over the past 2 decades, the mental health system in the Republic of Korea (hereafter Korea) has gone through several legal amendments. Before the 1990s, people with mental illness were often neglected at prayer centers or nursing homes without proper treatment [1]. Their human rights were often violated with oppressive treatment and dehumanizing environments. Treatment was merely custodial control, forcing patients to acquiesce. The first mental health law was enacted in 1995, to address mental illness prevention, treatment, and rehabilitation [1]. The law focused on accreditation of then unlicensed psychiatric facilities, as well as stipulating admission and discharge procedures in order to monitor and control the quality of psychiatric services.

Despite several minor revisions since then, infringement on patients’ human rights persists in many cases. As of 2016, the rate of involuntary hospitalization of mentally ill patients in Korea was above 60% [2], which is much higher than the average of other Organization for Economic Co-operation and Development (OECD) countries [3]. More than 51% of psychiatric inpatients were given no information about the hospitalization process, and about 35% of inpatients were secluded and restrained without any explanation [4]. Approximately 10% have experienced violence from hospital employees or other inpatients [4]. Long-term hospitalization in institutions has also been practiced. The average length of stay of psychiatric patients in Korea was the longest among OECD countries (233 days in 2008 and 116 days in 2011) [4, 5], marking Korea as a country with a high level of institutionalization [6].

Increasing awareness of the human rights of psychiatric patients has led to intensified efforts to protect those rights. In particular, in 2016, the Constitutional Court decided that the article of the Mental Health Act on involuntary admission by a legal guardian was unconstitutional. They ruled that it limited the patient’s physical freedom and lacked sufficient measures to prevent abuse [1].

In response to this, a substantial amendment (the Mental Health and Welfare Act; hereafter MHWL) was passed in May 2016 and took effect in May 2017. The revision focused on control of involuntary admissions through tightening of involuntary admission criteria. Under the revised MHWL, a patient who needs 2 weeks or more of involuntary admission must receive an additional cross-check of the diagnosis from another psychiatric specialist working at a national or public hospital [2]. After a patient is admitted, his or her case must be reviewed and approved by a designated committee within one month from admission to determine whether the hospitalization was appropriate [1, 7, 8]. For voluntary admissions, the period for reconfirmation of a patient’s voluntary intention to be treated was shortened from yearly to every two months [2]. Additionally, the MHWL revision stipulated that continuity of care after discharge be ensured by providing welfare services, including support of patients’s employment and habitation and coordination of appropriate mental health services from prevention to rehabilitation [2].

The MHWL revision did not intend deinstitutionalization to mean downsizing psychiatric beds, as in mid-twentieth century US and Europe. The basic goal of the revised act was to contribute to deinstitutionalization by shortening the length of stay, promoting discharge, and eventually helping patients return to their lives in the community and prevent re-admission; eventually, to ensure the human rights and autonomy of patients [9–11]. However, it is unclear whether the goals of the MHWL revision have been appropriately met in terms of deinstitutionalization and community-based mental health care management of patients.

Over the past half-century, several studies have been conducted to evaluate the effects of laws or regulations on deinstitutionalization [12–14]. Due to the heterogeneity of national contexts and regulations, previous studies have reported diverse results. Several studies have shown that strengthening hospitalization requirements may lead to a successful transition to community-based psychiatric services, including shortening the length of stay, increasing the utilization of community-based psychiatric services, and decreasing emergency room visits [15, 16]. One study showed that the shortened hospitalization period may cause increases in re-admission [17], whereas other studies showed that shorter hospitalization and re-admission rates were not related, reflecting inconsistent results [18, 19]. A recent study in Korea, using data from a small number of private mental hospitals, showed mixed results on the effects of the MHWL revision. The average length of stay decreased and the proportion of voluntary hospitalizations increased. However, the rate of re-admission increased, contrary to the purpose of the law [20].

Previous studies examining the effect of laws or regulations through descriptive comparisons of psychiatric service utilization before and after the laws have limitations; specifically, they did not have comparison groups or failed to control for secular trends in the data, concerning the potential for unmeasured confounding variables. Moreover, no study has examined the effects of the MHWL revision at the population level. Thus, this study was conducted to rigorously evaluate the effects of the revised MHWL on deinstitutionalization at the population level. Routinely collected data of hospital admissions and continuity of mental health service use after hospital discharge were used as proxy measures of deinstitutionalization. An interrupted time series (ITS) analysis, as a quasi-experimental research design, was applied.

## Methods

### Data source and measures

We used psychiatric medical utilization data at the population level from the National Health Information Database (NHID) from 2012 to 2019. The NHID is a public database on health care utilization, health screening, and mortality for the entire population of Korea (over 50 million) provided by the National Health Insurance Service [21]. From the health care utilization dataset, we used aggregated claims-based data on inpatient and outpatient service utilization claimed as schizophrenia (defined by International Classification of Diseases, 10th Revision codes F20.*-F29.*) as a principal or secondary diagnosis. These data were aggregated to identify trends and test the significance of events using ITS analysis.

We limited our population to schizophrenia patients who experienced non-voluntary hospitalization. They are more likely to have long-term stays than other psychiatric patients because the disease shows a degenerative and chronic course after controlling acute symptoms [22, 23]. We constructed our data to be suitable for ITS by calculating monthly measurements of variables for 96 months.

### Outcome variables: psychiatric patient service utilization

Deinstitutionalization is a complex and multifaceted phenomenon, the consequences of which cannot be measured by a single measurement [10]. Depopulation of mental hospitals requires discharges to exceed admissions. Thus, we used inpatient information on admissions and discharges. However, the process of deinstitutionalization cannot be fully measured by the total hospital population. With the primary objective of deinstitutionalization being to move the mentally ill from the hospital to the community, re-admission is a key indicator of whether the integration of discharged patients into the community has been successful [24]. If there are no significant changes in re-admission rates after the legislation, we can infer that integrating discharged patients from the hospital into the community has been successful [24]. The first admission rates were calculated by the number of first mental hospital admissions per 1,00,000 population. The discharge rates were calculated by discharges per inpatients within each month. We generated patient re-admission rates at 7, 30, and 90 days after discharge to examine short- and long-term trends in re-admissions. These rates were calculated based on re-admission cases falling within each of the relevant categories.

To investigate mental health service utilization changes in the community after discharge, we generated two outpatient variables based on an OECD Health paper [25]: (1) timely ambulatory follow-up within 7 and 30 days after discharge and (2) continuity of care defined as consistent visits at least once per month within 6 months after discharge. Timely ambulatory follow-up is critical for monitoring side effects that may result from inpatient medication changes and to support compliance with the treatment plan [25, 26]. Continuity of visits for 6 months after discharge has frequently been used to assess further recovery and prevent relapse [25, 27].

### Independent variable: MHWL revision

The MHWL was revised in June 2017. The period before the revision was coded as “0” and the period thereafter was coded as “1”.

### Statistical analysis

ITS analysis is arguably the strongest quasi-experimental research design, with a high degree of internal validity for evaluating the effectiveness of population-level health interventions implemented at a clearly defined point in time [28, 29]. Studies using observational data, including multivariate regression modeling, often fail to control for confounding variables and the difficulty in establishing causation, therefore using weak evidence to assess the effectiveness of an intervention or policy [30]. However, quasi-experimental study designs are able to estimate causal effects using observational approaches. ITS analysis is a useful quasi-experimental design for evaluating the longitudinal effects of interventions through regression modeling. This is especially true where cost or possible political or ethical concerns prevent randomization or the use of control groups [31]. This situation often happens when a national policy is implemented at a single time, making it impossible to have proper comparison groups. The approach requires constructing a time series of population-level data, taken repeatedly (typically at equal intervals) to test statistically for a change in the outcome rates in the periods before and after implementing a policy/program designed to change the outcome. The approach hypothetically sets a comparison (the counterfactual trend in the absence of the intervention), which is ‘interrupted’ by an intervention at a known point in time [32]. In the analysis, we estimated the changes in rate levels and trends after revising the law, controlling for baseline level and trend. Our model is below:

$$\mathrm{Y}=\mathrm{ \alpha }+ {\beta }_{1}T+ {\beta }_{2}X+ {\beta }_{3}TX+ \varepsilon$$

where *T* is time, *X* is the intervention phase, *TX* is time after the intervention, β_1_ is the pre-trend, β_2_ is the post-level change, β_3_ is the post-trend change, and β_1_ + β_3_ is the post-trend.

To improve the robustness of the analysis, we controlled for seasonality and autocorrelation. We tested seasonality in the time series data and the X-12-ARIMA method was used to adjust it [33]. To account for auto-correlated data, the ITS approach has employed autoregressive integrated moving-average models [34] or ordinary least squares (OLS) regression models designed to adjust for autocorrelation [35, 36]. We controlled for autocorrelation by fitting OLS regression models because they are often more flexible and broadly applicable in an interrupted time-series context [34, 37]. Our model estimated the coefficients using OLS regression and produced Newey-West standard errors to handle autocorrelation in addition to possible heteroskedasticity. To ensure that we fit a model that accounted for the correct autocorrelation structure, we conducted a post-estimation test using the Cumby-Huizinga test for autocorrelation [38]. All data were analyzed using Stata version 15.1 (Statan Corp LP, College Station, TX, USA). Ethical approval for this study was obtained from the Korean National Institute for Bioethics Policy (IRB #2019-1510-002).

## Results

Table 1 shows the estimated effects of the MHWL revision on both level and slope change in psychiatric patient service utilizations. Level change coefficients represent the immediate effect of changes in the service utilization after the revision and slope change coefficients represent the average rate of change per month.

**Table 1:** Results from interrupted time series analysis, with 2017 data

	Level change at the revision		Trend change after the revision	
	Coefficient	95% SE^a^	Coefficient	95% SE^a^
First admission (per 1,00,000 persons)	0.03	− 0.01 to 0.01	0.004	− 0.01 to 0.01
Discharge rates (%)	0.60	− 0.04 to 0.03	− 0.01	− 0.04 to 0.03
Readmission within 7 days (%)	− 2.24***	− 3.30 to − 1.18	− 0.10***	− 0.14 to − 0.05
Readmission within 30 days (%)	− 1.99*	− 3.72 to − 0.25	− 0.14***	− 0.22 to − 0.07
Readmission within 90 days (%)	− 0.01	− 0.03 to 0.01	− 0.001*	− 0.002 to − 0.0002
Outpatient visit within 7 days (%)	1.98**	0.68–3.28	− 0.06	− 0.12 to 0.01
Outpatient visit within 30 days (%)	2.72*	0.22–5.21	− 0.05	− 0.17 to 0.08
Continuous care (%)	4.00*	0.31–7.69	− 0.27	− 0.63 to 0.08

^a^Newey-West standard errors

^*^p  <  0.05, **p  <  0.01, ***p  <  0.001

**Fig. 1:**
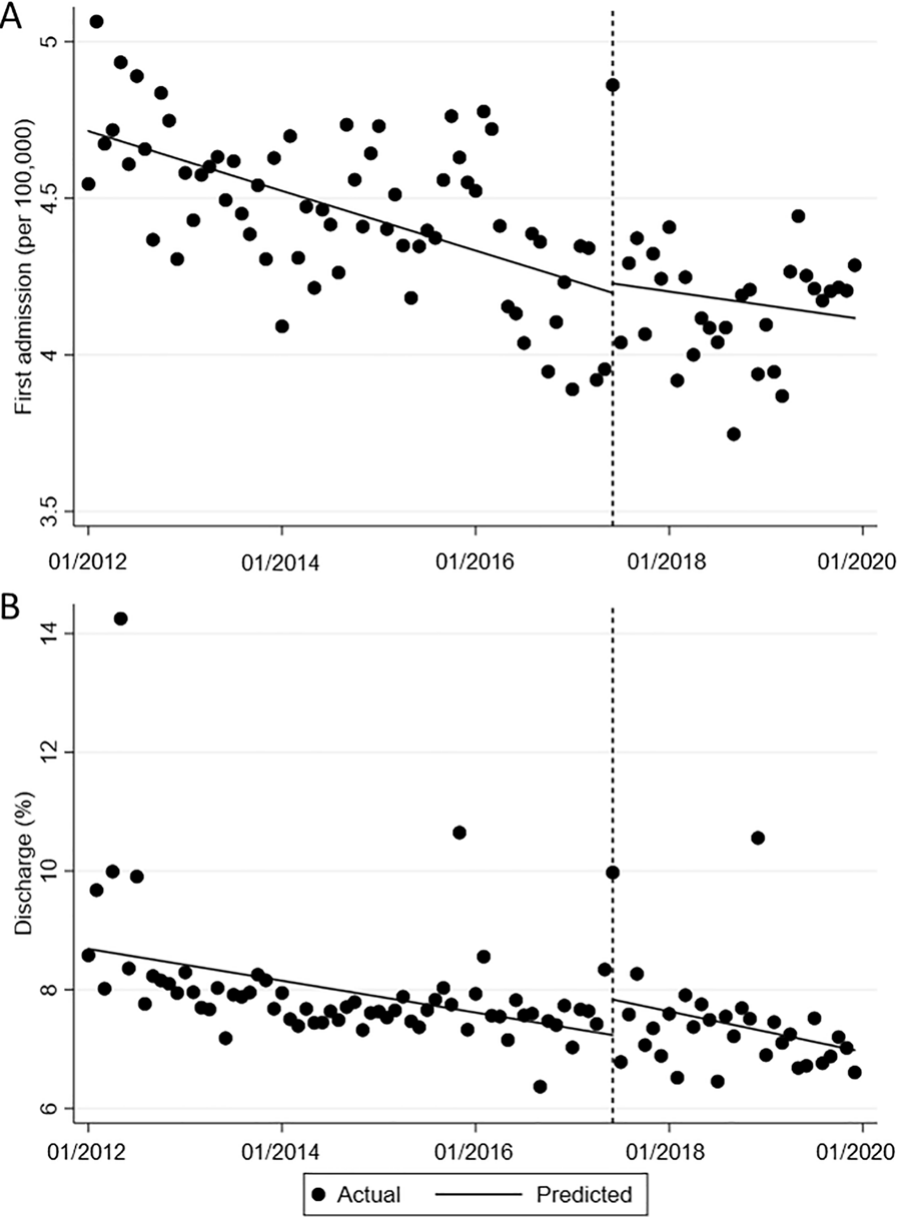
Interrupted time series plot of the monthly change in first admission (**A**) and discharge rates (**B**). The vertical line represents June 2017 when the Mental Health and Welfare Act was revised, the dots represent monthly points, and the slope is the regression line derived from time series analysis

Before the revision of the law, the first admission (incidence) and discharge rates showed decreasing rates, at 0.008 and 0.02 persons per month, respectively (Table 1). There was no statistically significant change in either level or slope in the first admission (incidence) and discharge rates after the revision (Fig. 1).

**Fig. 2:**
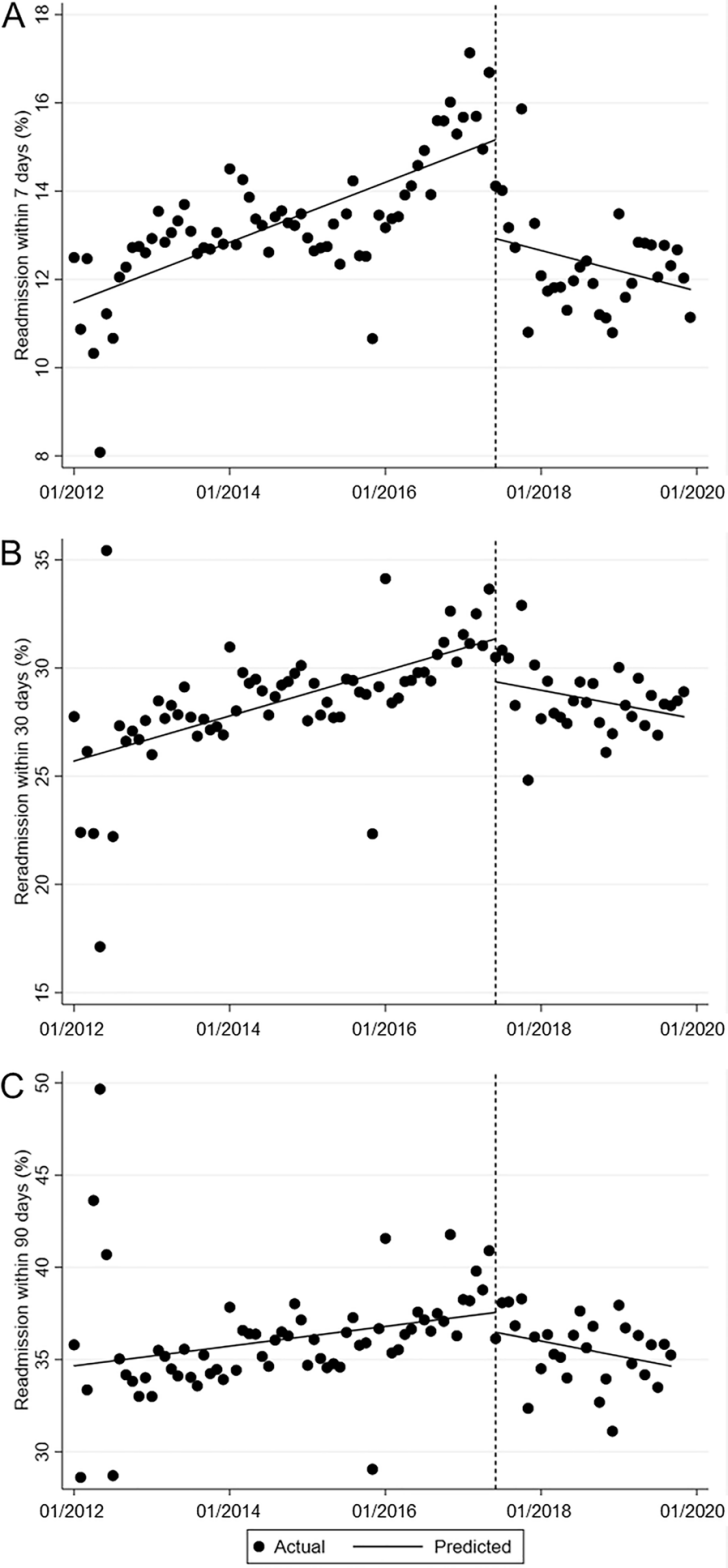
Interrupted time series plot of the monthly change in re-admissions within 7 days (**A**), 30 days (**B**), and 90 days (**C**) after discharge. The vertical line represents June 2017 when the Mental Health and Welfare Act was revised, the dots represent monthly points, and the slope is the regression line derived from time series analysis

The key variable for deinstitutionalization, changes in re-admission rates, showed an increasing secular trend before the revision, but showed decreasing trends after the revision (Fig. 2). Before the law revision, the rates of re-admission within 7 and 30 days showed increasing trends, at 0.15% and 0.09% per month, respectively. The rates of re-admission within 90 days showed increasing trends, at a rate of 0.0004% per month. The levels of re-admission within 7 and 30 days decreased by 2.24% and 1.99% subsequent to the revision, respectively (*p*  <  0.001; *p*  <  0.05). The slopes of the monthly trends of re-admission within 7 and 30 days decreased by 0.10% and 0.14%, respectively (*p*  <  0.001). For the rate of re-admission within 90 days, a significant decrease was only found for the slope (0.001%) (*p*  <  0.05).

**Fig. 3:**
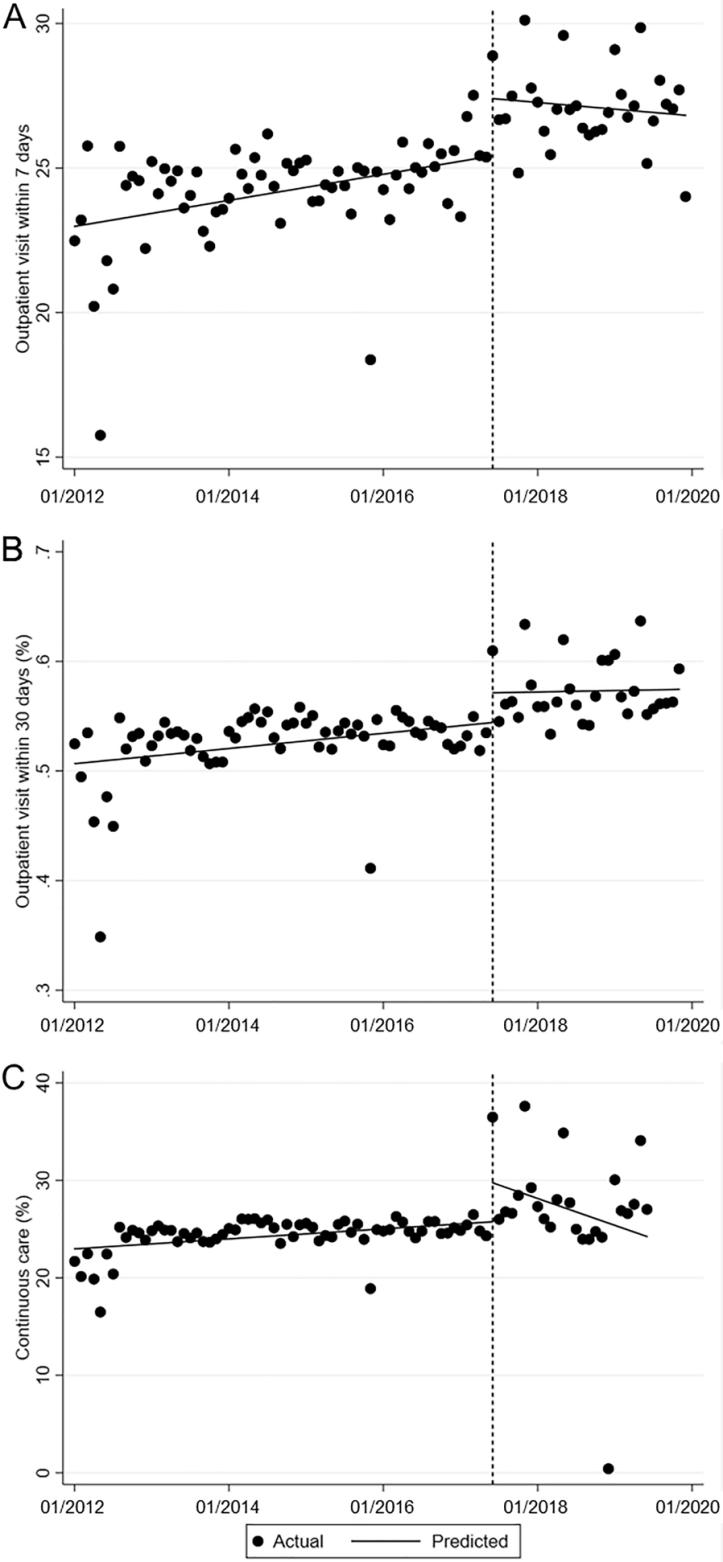
Interrupted time series plot of the monthly change in outpatient visits within 7 days (**A**), 30 days (**B**), and continuous care (**C**) after discharge. The vertical line represents June 2017 when the Mental Health and Welfare Act was revised, the dots represent monthly points, and the slope is the regression line derived from time series analysis

In contrast, after the law revision, the level changes in monthly rates of outpatient services among those who were discharged significantly increased (Fig. 3). The level changes in monthly rates of outpatient visits within 7 and 30 days showed increases of 1.98% and 2.72%, respectively (*p*  <  0.01, *p*  <  0.05). The level change of monthly rates of continuous care showed a significant increase of 4.0% (*p * <  0.05). However, the slope changes in the rates of outpatient services did not differ significantly after the revision.

## Discussion

This study is the first attempt to evaluate the effects of the MHWL revision, which tightened the involuntary admission criteria on deinstitutionalization among schizophrenia patients in Korea. We obtained two salient findings. First, our study found an immediate slight drop and a downward trend in the re-admission rates, despite no statistically significant changes in the first admission and discharge rates. Second, the immediate increase in outpatient visits was significant, whereas the slope changes in outpatient visits did not change significantly after the revision.

Although the revision introduced additional regulations for involuntary admissions and more frequent reconfirmation of a patient’s voluntary intention to be treated, our study showed no statistically significant changes in the first admission and discharge rates after the revision. This finding implies that the revision did not disrupt the first admissions or facilitate discharges as a whole. Involuntary first admissions may have already gradually decreased. This argument is supported by the fact that only 1% of the first admissions were identified as inappropriate by committees evaluating the appropriateness of hospitalization [39]. Alternatively, long-term inpatients who were admitted involuntarily were substituted for voluntary admissions before the revision. Private psychiatric hospitals may have substituted involuntary admissions of long-term inpatients with voluntary admissions by obtaining consent from patients and families before the revision, as most do not have proper alternative psychiatric institutions in their communities [40].

Our study showed that the re-admission rates within 7 and 30 days were immediately lower and showed downward trends after the revision in the absence of significant changes in the first admissions and discharges. A recent study analyzing medical records from two Korean psychiatric hospitals showed an increase in re-admission rates after the revision [20]. However, that study is limited the generalizability of the study findings and is not compatible with our study because all patients were included in that study, regardless of diagnosis. Our study findings may be explained by the effect of the strict requirements for long-term involuntary admission. For involuntary re-admissions, it is necessary to prove that the patient poses a threat of both self-harm and other-harm. This tight regulation requires the patients to be exacerbated or relapsed enough to express the danger of self-harm and other-harm. In contrast, Western countries require a condition of either self-harm or other-harm for involuntary admission [41]. Thus, the decrease of re-admission may be a negative signal that patients with recurrence or worsening of psychosis cannot be readmitted when required, especially within a short period after discharge.

Conversely, it can be seen as a positive effect that the revision contributed to deinstitutionalization. This argument can be supported by our study finding that outpatient use and continuous care of discharged patients increased significantly after the revision. However, our study also shows that the magnitude of effects on re-admission decreased over time after discharge, and there was no significant change in trends of outpatient service use. This finding may imply that the revision was slightly effective for integrating discharged patients from hospitals into their communities [24]; however, there was not enough preparation and infrastructure to provide adequate protection and care for the patients in the community. The purpose of deinstitutionalization is to reduce involuntary hospitalization and allow discharged patients, at their will, to use mental health care based on their needs within their community. In particular, in the early stages of discharge, management through regular case manager visits was reported to play an important role in their stable living [42, 43].

In Western countries where deinstitutionalization efforts were made earlier, there was also an emphasis on providing sufficient alternative home and community-based services to replace treatment during hospitalization [9, 44]. Following the revised law, Mental Health and Welfare centers were established in every district in Korea to take on this role in the community. Various tasks, including case management of registered mentally ill people in the community, day-care services, education and training, linkage with other institutions, and mental health promotion programs are carried out in these centers [2]. However, due to the lack of skilled workers and limited budgets, sufficient management for the rehabilitation of people with severe mental illness is limited [45].

Our study has several limitations. First, examining the effect of the revision on changes in involuntary re-admissions was not possible because the national claims data did not provide information on whether an admission was involuntary or not. This information cannot be obtained from administrative data from the Department of Health and Welfare, the department in charge of mental health policy. Second, we were not able to explore medical service utilization at an individual level because our analysis was based on a single set of time series data, not a form of cohort data at an individual level. Thus, we were not able to examine the trajectories of psychiatric hospital service use among patients with schizophrenia and its relationships to length of stay, discharge, outpatient service use, and re-admission. Lastly, our study cannot exclude the possibility of drop-out in the data from schizophrenia patients who were homeless or incarcerated. In countries such as the United States and the United Kingdom, side effects of deinstitutionalization have been reported, such as an increased number of homeless people with mental illness or an increase in patients with mental illness in prisons and nursing homes. There have also been cases in which many patients become more seriously isolated and desperate because they are unable to participate in community activities after discharge [9]. There have also been attempts to empirically analyze the Penrose hypothesis, according to which the rate of mental hospital use and the number of prisoners in prison are inversely proportional [46, 47].

## Conclusion

Our study showed that the revision led to a significant, but slight increase in re-admissions and decreased outpatient visits among schizophrenia patients. The study focused on the effects of the law revision at an early stage. Future studies need to be conducted to monitor and evaluate whether the revision has led to successful deinstitutionalization. Furthermore, studies using individual-level follow-up data would be advantageous to examine how the trajectories of psychiatric service use among patients with schizophrenia have changed and eventually whether their quality of life and mental health has improved in the long run. Our study suggests that strengthening hospitalization requirements may help to decrease re-admissions and aid transit to community-based psychiatric services among schizophrenia patients in the short period. Long-term monitoring and evaluation are needed for successful deinstitutionalization, given the heterogeneity of national contexts and regulations.

## Data Availability

The datasets used for the study are available from the corresponding author on reasonable request.

## Abbreviations

MHWL: Mental Health and Welfare Act
NHID: National Health Information Database
ITS: Interrupted time series
OLS: Ordinary least squares
